# What Acute Stress Protocols Can Tell Us About PTSD and Stress-Related Neuropsychiatric Disorders

**DOI:** 10.3389/fphar.2018.00758

**Published:** 2018-07-12

**Authors:** Laura Musazzi, Paolo Tornese, Nathalie Sala, Maurizio Popoli

**Affiliations:** Laboratory of Neuropsychopharmacology and Functional Neurogenomics – Dipartimento di Scienze Farmacologiche e Biomolecolari and Center of Excellence on Neurodegenerative Diseases, University of Milano, Milan, Italy

**Keywords:** animal models of mental disorders, stress disorders, post-traumatic, ketamine, synaptic morphology, glutamic acid, vulnerability, resilience

## Abstract

Posttraumatic stress disorder (PTSD), the fifth most prevalent mental disorder in the United States, is a chronic, debilitating mental illness with as yet limited options for treatment. Hallmark symptoms of PTSD include intrusive memory of trauma, avoidance of reminders of the event, hyperarousal and hypervigilance, emotional numbing, and anhedonia. PTSD is often triggered by exposure to a single traumatic experience, such as a traffic accident, a natural catastrophe, or an episode of violence. This suggests that stressful events have a primary role in the pathogenesis of the disorder, although genetic background and previous life events are likely involved. However, pathophysiology of this mental disorder, as for major depression and anxiety disorders, is still poorly understood. In particular, it is unknown how can a single traumatic, stressful event induce a disease that can last for years or decades. A major shift in the conceptual framework investigating neuropsychiatric disorders has occurred in recent years, from a monoamine-oriented hypothesis (which dominated pharmacological research for over half a century) to a neuroplasticity hypothesis, which posits that structural and functional changes in brain circuitry (largely in the glutamate system) mediate psychopathology and also therapeutic action. Rodent stress models are very useful to understand pathophysiology of PTSD. Recent studies with acute or subacute stress models have shown that exposure to short-time stressors (from several minutes to a few hours) can induce not only rapid, but also sustained changes in synaptic function (glutamate release, synaptic transmission/plasticity), neuroarchitecture (dendritic morphology, synaptic spines), and behavior (cognitive functions). Some of these changes, e.g., stress-induced increased glutamate release and dendrite retraction, are likely connected and occur more rapidly than previously thought. We propose here to use a modified version of a simple and validated protocol of footshock stress to explore different trajectories in the individual response to acute stress. This new conceptual framework may enable us to identify determinants of resilient versus vulnerable response as well as new targets for treatment, in particular for rapid-acting antidepressants. It will be interesting to investigate the putative prophylactic action of ketamine toward the maladaptive effects of acute stress in this new protocol.

## Stress-Related Neuropsychiatric Disorders: PTSD

One day a person experiences deep stress caused by a sudden incident (a traffic accident, a natural catastrophe, an episode of violence). Subsequently, this person could develop a serious neuropsychiatric disorder, posttraumatic stress disorder (PTSD), that may last for years or even decades. Hallmark symptoms of PTSD include intrusive memory of the traumatic event, avoidance of reminders of the event, hyperarousal and hypervigilance, emotional numbing, and anhedonia (Kessler et al., 1995; American Psychiatric Association [APA], 2013).

Stress is considered a primary risk factor for most neuropsychiatric disorders, including depression, anxiety, and PTSD (Duman and Aghajanian, 2012; Pitman et al., 2012; Popoli et al., 2012; McEwen et al., 2015; Duman et al., 2016; Murrough et al., 2017). The latter is probably the single disorder where the relationship with traumatic stress is more evident. Indeed, although not always this applies, PTSD is often induced by a single (acute) traumatic experience.

Posttraumatic stress disorder is a chronic and debilitating illness with as yet limited options for treatment (Berger et al., 2009; Krystal et al., 2017b). A main reason for this problem is the still poor insight we have into the pathogenetic mechanism of disease (Gordon et al., 2017). However, a large body of clinical research has investigated the changes observed in brains of people affected by PTSD by using neuroimaging techniques.

## Neuroimaging Studies of PTSD

**FIGURE 1:**
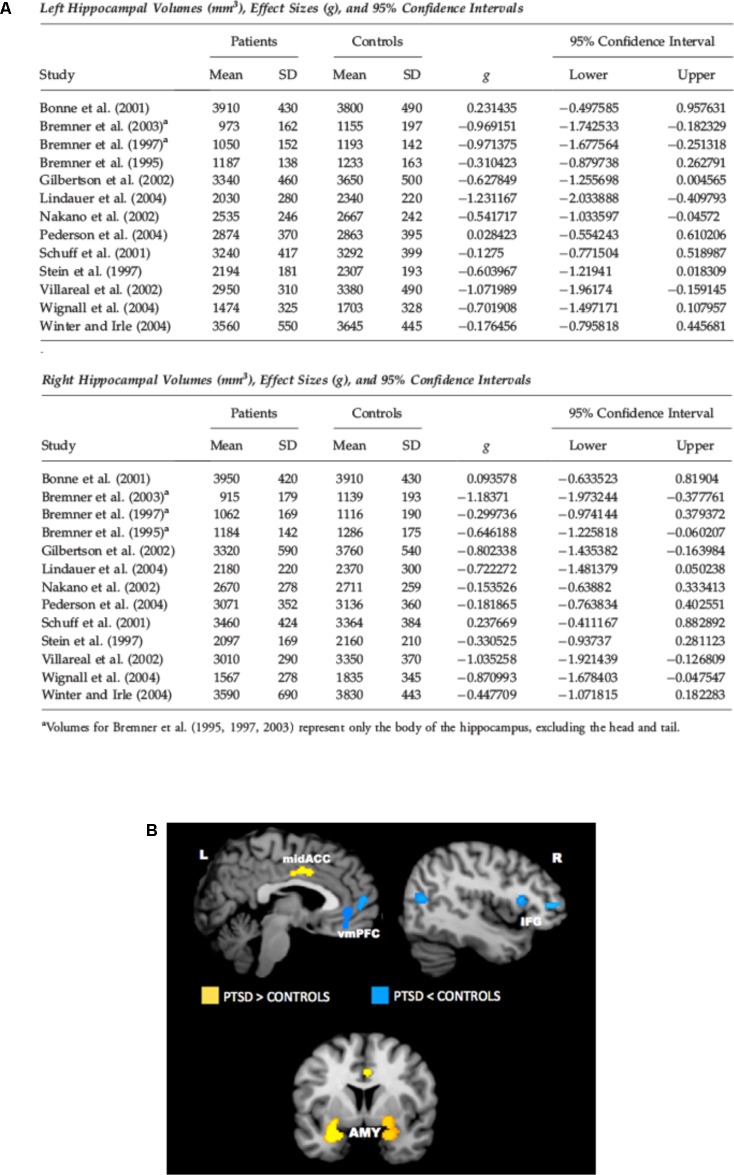
**(A)** Meta-analysis of studies in which hippocampal volume was estimated from magnetic resonance images in adult patients with PTSD. The meta-analysis included 13 studies of adult patients with PTSD that compared the patients to well-matched control groups (total 215 patients and 325 control subjects). Pooled effect size calculations across the studies indicated significant volume differences in both hemispheres. On average PTSD patients had a 6.9% smaller left hippocampal volume and a 6.6% smaller right hippocampal volume compared with control subjects. These volume differences were smaller when comparing PTSD patients with control subjects exposed to similar levels of trauma, and larger when comparing PTSD patients to control subjects without significant trauma exposure. Reproduced with permission from Smith (2005). **(B)** Meta-analysis of functional neuroimaging studies in PTSD. Across symptom provocation and cognitive-emotional tasks, the amygdala and mid-ACC are hyperactive in PTSD whereas the lateral and medial prefrontal cortex are hypoactive for negative emotional stimuli vs. neutral and positive stimuli. Areas of hyperactivation in PTSD (PTSD > Control) are shown in yellow, areas of hypoactivation in PTSD (Control > PTSD) are shown in blue. AMY, amygdala; IFG, inferior frontal gyrus; L, left; mid-ACC, mid anterior cingulate cortex; R, right; vmPFC, ventromedial prefrontal cortex. Reproduced with permission from Hayes et al. (2012a).

There is a wide literature in the last two decades of both structural and functional neuroimaging studies in PTSD. Magnetic resonance imaging (MRI) studies found lower volume of hippocampus (HPC), rostral ventromedial prefrontal cortex (vmPFC), and dorsal anterior cingulate cortex (dACC) in PTSD (Bremner et al., 1995; Gurvits et al., 1996; Smith, 2005; Kitayama et al., 2006; Kasai et al., 2008; O’Doherty et al., 2015) (see **Figure 1A** for HPC). Functional neuroimaging studies using functional MRI or Positron Emission Tomography (PET) found altered activity in amygdala, vmPFC, dACC, HPC, and insular cortex. In particular, amygdala (which is powerfully activated by stressful events and has a crucial role in fear learning), dACC, and insular cortex were hyperactivated in subjects with PTSD. Conversely, vmPFC showed decreased activation, while HPC showed less activation in some studies and more activation in others (**Figure 1B**). Decreased vmPFC activity was associated with increased amygdala activity. Many of these studies (both structural and functional) have been the object of meta-analyses, substantially confirming all these alterations (Karl et al., 2006; Kitayama et al., 2006; Hayes et al., 2012a; O’Doherty et al., 2015). These results are in line with neurocircuitry models of PTSD, which posit that reduced top-down control of amygdala by vmPFC is implicated in PTSD symptomology, with increased attentional bias toward threat, increased fear response, and defective extinction of traumatic memories (Rauch et al., 2006; Pitman et al., 2012). Some of the changes detected by neuroimaging in PTSD are reminiscent of similar features found for depressed brains, which poses the question of what could be the biological markers of two distinct pathologies that have clear different simptomology, but also a wide area of comorbidity (Shalev et al., 1998).

In all the brain areas above glutamatergic neurons and synapses are largely predominant. In the neocortex, representing 85% of the human brain, about 80% of the neurons are excitatory, most use glutamate as neurotransmitter and form about 85% of all synapses (Orrego and Villanueva, 1993; Douglas and Martin, 2007; Nava et al., 2015). A number of studies in recent years analyzed in detail the role of stress and stress-related molecular/cellular/functional effects in the glutamate system, in pathophysiology of neuropsychiatric disorders (Gorman and Docherty, 2010; Duman and Aghajanian, 2012; Popoli et al., 2012; Sanacora et al., 2012; McEwen et al., 2015; Averill et al., 2017; Musazzi et al., 2017). Within this framework, a large body of evidence on the role of the glutamate system comes from studies with rodent models of stress (see below).

## The Role of Environmental Stimuli in PTSD: Open Questions

A crucial question is whether some components in the complex PTSD symptomology are pre-existent at the time of trauma and may be responsible for susceptibility of some individuals. Because a minor (although remarkable) portion of the population exposed to a traumatic stressor develops the disorder, it is generally assumed that genetic background and previous life events contribute to inducing a pro-adaptive (resilient) or maladaptive (vulnerable) stress response to the traumatic event (Galea et al., 2005; Horn et al., 2016; Musazzi et al., 2017). This is of course not dissimilar from other neuropsychiatric disorders, yet the case for PTSD is emblematic because it clearly shows that a number of subjects exposed to the same acute traumatic event can display great individual variability in the long-term response to the same trauma (Yehuda, 1999; Daskalakis et al., 2013).

The defining symptoms of PTSD include: re-experiencing over time the traumatic event, a feature that has been related to defective extinction of fear memory; high anxiety and hyperarousal, a feature related to a general sensitization of the nervous system (Hayes et al., 2012b; Pitman et al., 2012; Bennett et al., 2016; Horn et al., 2016). Studies with identical twins discordant for combat exposure (one was a Vietnam veteran and the other was not exposed to combat experience) found that only PTSD-affected twins showed defective fear extinction measured as conditioned skin conductance response (Milad et al., 2008). Similarly, studies on increased nervous system sensitization, measured as heart-rate responses to loud tone stimuli, strongly suggested that heightened heart-rate reactivity in PTSD-affected twins is an acquired feature of the disorder (Orr et al., 2003; Metzger et al., 2008). While these studies did not exclude a role for genetic predisposition (or previous life events) in PTSD, they showed that the environmental stimulation has a primary role in this disease. On the other hand, other human studies showed that individual differences in some of these parameters before trauma exposure may be predictors of severity of PTSD symptoms after trauma (Guthrie and Bryant, 2006; O’Donnell et al., 2007; Orr et al., 2012).

Indeed, one of the main questions about PTSD is still waiting for an answer: how can a single, although traumatic, stressful event induce a disease that can last for years or decades? A second, also crucial, question is: what differentiates the maladaptive stress response of some individuals from the pro-adaptive response of the majority of subjects? A main goal of this article will be to report lines of evidence that may help addressing these questions. Tackling the multiple biological factors (and their interaction), that result in resilience or vulnerability over the stress response, is a main task for research aiming at understanding pathophysiology of PTSD and finding new targets for better, more efficient therapy (Gordon et al., 2017; Krystal et al., 2017b). Within this framework, rodent models of stress have a crucial role in research on PTSD (Daskalakis et al., 2013; Flandreau and Toth, 2017).

## Stress Protocols and Models of Psychopathology: PTSD

There is a rich literature on stress protocols in rodents, often used as models of psychopathology. While different models have been used to look at the biological bases of distinct neuropsychiatric disorders, no particular model can individually be related to a single pathology, for the obvious reason than no animal model can entirely reproduce psychopathology of human brain (Bennett et al., 2016; Musazzi et al., 2017; Slattery and Cryan, 2017). In general, most traditional animal models for these disorders are based on protocols of repeated or chronic stress. However, the peculiar nature of PTSD, that is often triggered by a single strong traumatic event, has suggested essential requirements for a model of PTSD to have good translational value: (1) the trauma must be relatively severe, (2) a short duration of protocol should be sufficient to provoke PTSD-like symptoms, (3) the intensity of the trauma should predict the severity of outcome, and (4) significant interindividual variability should be observed in outcomes (Siegmund and Wotjak, 2006; Flandreau and Toth, 2017). A number of stress protocols inducing PTSD-like symptoms are listed in **Table 1**. It is not within the scope of this article to review in detail the different stress protocols that have been used; the reader may find accurate description of the protocols in the reviews cited above and therein. Instead, we would like to draw attention to a few studies that have recently shown how acute stress may induce both short-term and long-term consequences in both functional and structural features of brain synapses and circuitry, which may be of help to understand how acute stress affects the extended stress response (Musazzi et al., 2017).

**Table 1:** PTSD rodent models.

Escapable/inescapable electric shocks	Siegmund and Wotjak, 2006
Predator/predator scent exposure	Roth et al., 2011
Single prolonged stress	Souza et al., 2017
Restraint/immobilization	Mitra et al., 2005
Underwater holding	Richter-Levin, 1998
Social defeat	Golden et al., 2011

## The Sustained Effects of Acute Stress on Synaptic Function and Morphology

Previous studies have investigated the short-term effect of a standard footshock (FS) stress protocol on glutamate release/transmission in PFC (a brain area shown to be less functional in PTSD) of rats. The acute inescapable FS protocol is widely used as a model to induce long-term behavioral changes resembling PTSD, and has the advantage to allow precise and reproducible control of the shock parameters, as well as clearly defined and reproducible context for stress (Bali and Jaggi, 2015; Flandreau and Toth, 2017). Among long-term behavioral changes induced by FS are: social avoidance, defensive behavior, hypervigilance, sleep disturbances, generalization of fear (Flandreau and Toth, 2017).

**FIGURE 2:**
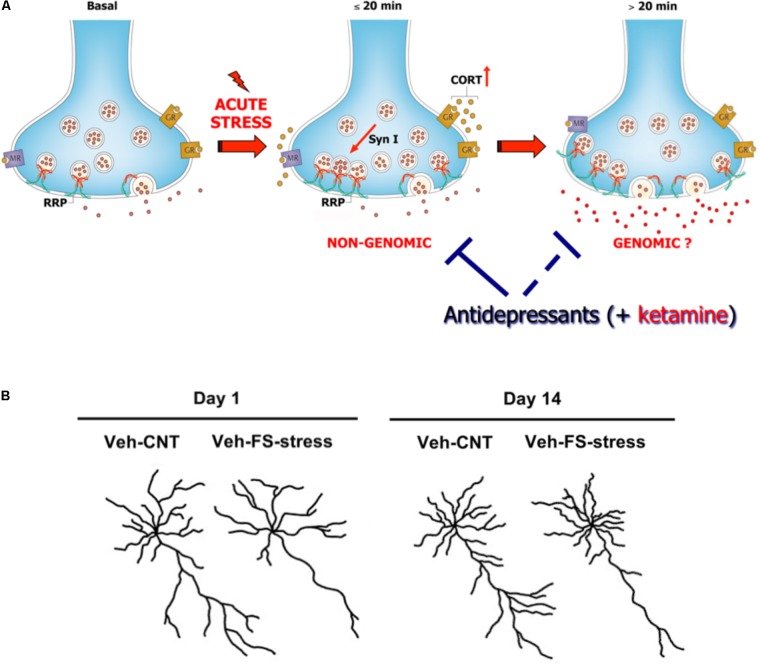
**(A)** Acute stress increases circulating levels of CORT that binds to local synaptic mineralocorticoid/glucocorticoid receptors. This in turn induces, in synaptic terminals of prefrontal and frontal cortex, a rapid increase of vesicle trafficking into the readily releasable pool (RRP) of glutamate vesicles, with consequent increase of RRP size (<20 min). This implicates non-genomic mechanisms, in particular phosphorylation of the presynaptic protein synapsin I at Ser^9^. The increase of RRP size, rapidly induced by CORT, primes the terminals for the subsequent enhancement of depolarization-evoked glutamate release. This was shown by *in vitro* incubation of purified synaptic terminals (synaptosomes) with CORT, which replicates the increase in the size of glutamate RRP and synapsin I phosphorylation. Slower (>20 min), perhaps partly genomic, effects are required to promote the enhancement of glutamate release and excitatory transmission induced by acute stress. The acute stress-induced enhancement of glutamate release is blocked by previous chronic treatment with antidepressants (but also by acute ketamine treatment, see below). Antidepressants block the buildup of RRP, but likely also act on subsequent events leading to glutamate release enhancement. Modified from Treccani et al. (2014b). **(B)** Representative reconstructions of prelimbic pyramidal neurons. Length of apical dendrites in layer II/III prelimbic PFC was significantly reduced 1 and 14 days after acute FS stress. Veh-CNT, control animals; Veh-FS-stress, FS stressed animals. Modified from Musazzi et al. (2017).

Acute FS stress exerts a destabilizing effect on glutamate transmission, rapidly enhancing glutamate release and excitatory synaptic transmission in PFC, an effect prevented by chronic antidepressant treatments. This was shown by using measurement of exocytotic release of glutamate from purified superfused PFC synaptic terminals (synaptosomes), and patch-clamp recordings (Musazzi et al., 2010; Popoli et al., 2012). The short-term synaptic effect of stress was mainly due to corticosterone (CORT) binding to synaptic receptors and rapid (non-genomic) enhancement of trafficking of glutamate presynaptic vesicles into the readily releasable pool (RRP), in perforated and non-perforated excitatory synapses, dependent on phosphorylation of the presynaptic protein synapsin I at Ser^9^ (Treccani et al., 2014a; Nava et al., 2015; **Figure 2A**). At the same time, acute FS stress dramatically increased the total number of non-perforated synapses in PFC, an effect again partly prevented by chronic antidepressants (Nava et al., 2015).

**FIGURE 3:**
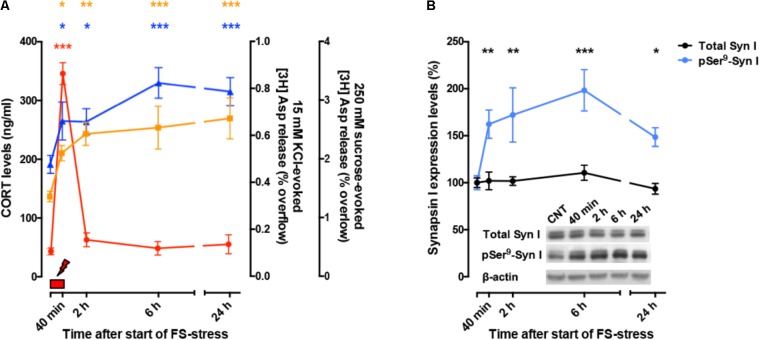
**(A)** Time course of CORT serum levels (red), 15 mM KCl-evoked [3H]D-Asp overflow (blue), and 250 mM sucrose-evoked [3H]D-Asp overflow (yellow) from superfused PFC/FC synaptosomes, after acute FS stress. The net depolarization-evoked overflow was calculated by subtracting transmitter content of the basal outflow. 15 mM KCl-evoked overflow is a measure of depolarization-evoked glutamate release; 250 mM sucrose-evoked overflow is a measure of RRP. The timing of actual FS stress (40 min) is indicated by the red mark. **(B)** Time course of total synapsin I expression and phospho-Ser^9^ (site 1) levels in synaptic membranes from PFC/FC after acute FS stress. Data are expressed as mean ± s.e.m. Insets: representative immunoreactive bands. **(A,B)** Modified from Musazzi et al. (2016).

So far, these studies showed that acute stress has rapid, destabilizing effects on glutamate synaptic transmission (**Figure 2A**). However, the picture became more complex when it was found that significant atrophy and remodeling of apical dendrites was observed in PFC as early as 24 h after FS stress. This was unexpected because stress-related dendritic remodeling, a consistently found outcome of stress in rodents (considered a key biological correlate of stress-related pathology in humans), is normally observed after chronic stress (Duman and Aghajanian, 2012; Sanacora et al., 2012; Sousa and Almeida, 2012; Musazzi et al., 2013). Moreover, the significant changes in apical dendrites were not transient but were observed for (at least) 2 weeks after acute FS stress (Nava et al., 2017; **Figure 2B**). These results clearly showed for the first time that a brief episode of inescapable stress may induce long-term neuroarchitectural changes in the brain. A widely shared hypothesis suggests that abnormal enhancement of glutamate release and excitatory transmission induced by stress results in dendritic atrophy/remodeling in PFC, particularly if this is repeated or sustained over time (Gorman and Docherty, 2010; Duman and Aghajanian, 2012; Sanacora et al., 2012; Musazzi et al., 2013; Abdallah et al., 2015; McEwen et al., 2015). Therefore, next we investigated whether the enhancement of glutamate release induced by acute stress was transient or sustained. As expected, CORT serum levels rised rapidly during the acute FS stress protocol but were back to nearly physiological levels within 2 h. Instead, depolarization-evoked glutamate release, after its rapid rise during FS stress, remained enhanced for at least 24 h in PFC after application of the stressor. RRP size, which is functional to the enhancement of glutamate release was also increased for at least 24 h (Musazzi et al., 2016). Even the phosphorylation of Ser^9^ of synapsin I, previously shown to be essential to the RRP size increase (Treccani et al., 2014a), was still way up at 24 h (**Figure 3**).

Taken together, these studies showed that acute stress may induce not only rapid structural and functional changes in glutamate synapses/circuitry but also result in sustained remodeling of neuroarchitecture. It is conceivable that the prolonged enhancement of glutamate release induced by acute stress triggers the retraction of apical dendrites, in an attempt to reduce the possible excitotoxic effect of glutamate. This is probably an adaptive physiological mechanism, not dissimilar from that observed in the brain of hibernating mammals (for a discussion see: Magariños et al., 2006; Musazzi et al., 2017). Although dendrite remodeling (and loss of synaptic spines) observed after chronic stress protocols are considered maladaptive changes, with acute stress it is difficult to distinguish between pro-adaptive and maladaptive changes. Remodeling of dendrites as a protective strategy may possibly turn into maladaptive changes, depending on several components, including genetic background, previous adverse life events, and nature/intensity/length of the stressful event; currently too little is known about the long-term response to acute stress and more work is required (see below).

A few previous studies analyzed the effects of acute stress, or a few closely spaced stressors, on neuroarchitecture. Izquierdo et al. (2006) found that mice exposed to a single session or three sessions of forced swim stress on consecutive days displayed shorter branching of apical dendrites of pyramidal neurons in infralimbic PFC. In a different study Hajszan et al. (2009) found that a standard learned helplessness (LH) protocol, a popular model of depression based on two FS stress sessions spaced 24 h, with the second one including active avoidance of the shock, induced loss of synaptic spines in rat HPC 1 and 7 days later (although dendritic development was not measured). Later studies analyzed the effects of short multimodal stress, lasting up to 5 h (Chen et al., 2010). The authors found that this brief but complex stress protocol induced a deficit in cognitive ability, associated with disruption of long-term potentiation and reduction of spines in hippocampal CA3 area. The degree of memory deficit in individual mice correlated significantly with the reduced density of area CA3 apical dendritic spines. These and later additional experiments suggested that CORT and corticotropin-releasing hormone (CRH), via their cognate receptors, act sinergistically on RhoA, a spine-actin regulator, to promote its deactivation and degradation and consequently destabilize synaptic spines. Exposing hippocampal slices concomitantly to stress-equivalent levels of CORT and CRH for at least 1 h recapitulated physiological, structural, and behavioral effects of stress (Chen et al., 2016). These molecular changes could represent a pathway whereby acute/subacute stress modifies neuroarchitecture.

## The Link Between Acute Stress and Psychopathology

**FIGURE 4:**
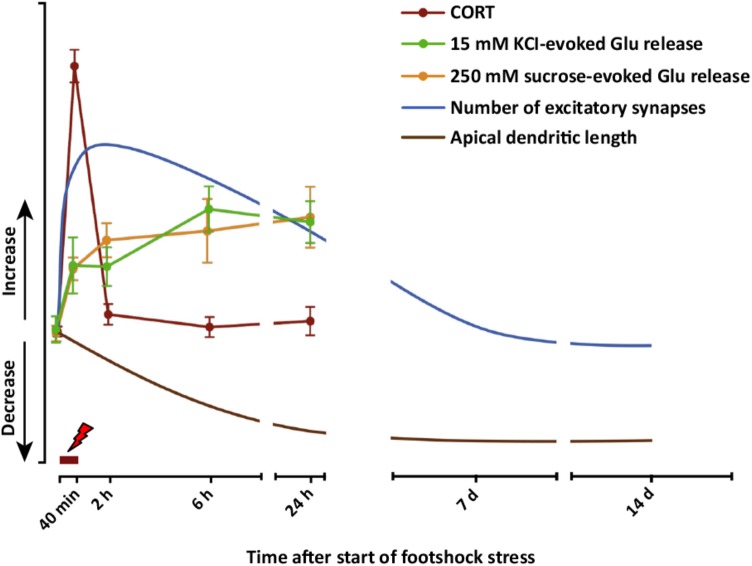
Graphic summary of short- and long-term functional and neuroarchitectural effects in prefrontal cortex (PFC) synapses after acute FS stress. The fast and transient increase in CORT release induced by acute FS stress was accompanied by the rapid increase in both depolarization-evoked and hypertonic sucrose-evoked (RRP) glutamate release in PFC, and the number of small excitatory synapses. The enhancement of glutamate release was sustained for up to 24 h, as well as the increased number of excitatory synapses, which normalized between 24 h and 7 days after FS. Before 24 h had elapsed from the start of FS stress, retraction of apical dendrites began and was sustained for up to 14 days. The timing of actual FS stress (40 min) is indicated by the red mark. Number of excitatory synapses and apical dendrite length are indicative and not in scale with other readouts. From Musazzi et al. (2017).

Taken together, these results obtained with acute or subacute stress protocols change the traditional distinction operated between the effects of acute versus chronic stress. The results show that stress exposure restricted to a duration of minutes or hours may have long-term structural (dendrite atrophy/loss of spines), functional (glutamate release/synaptic transmission and plasticity), and behavioral (cognitive functions) consequences. These sustained stress-related changes suggest a mechanism whereby acute stress affects the extended stress response, and may contribute to explain how limited exposure to acute traumatic stress results in pathogenesis of stress-related disorders, including PTSD. A complete dynamic dissection of the short- and long-term effects of acute stress is required to understand when and how the stress response (a physiological brain and body response) turns into pathology-related maladaptive changes. **Figure 4** graphically summarizes several short- and long-term functional and neuroarchitectural effects induced by acute FS stress in glutamate synapses and circuitry in PFC, reporting also the fast transient peak of CORT during and right after the stress protocol (from Nava et al., 2015, 2017; Musazzi et al., 2016). The fast rise of glutamate release during and right after FS stress is mainly mediated by the action of CORT at synaptic receptors and by the increase in the RRP of glutamate in presynaptic terminals (as shown in Treccani et al., 2014a). The subsequent sustained enhancement of glutamate release during the first 24 h is accompanied by retraction of apical dendrites in infralimbic PFC, which was first measured at 24 h and then up to 2 weeks. This circumstantial evidence is in line with the hypothesis that excessive and sustained glutamate release, consequent to FS stress, is a main reason for dendritic remodeling, but does not provide complete evidence of a causal relationship. *In vitro* experiments with primary neuronal cultures treated with CORT will explore in detail this hypothesis.

However, there is compelling evidence in the literature that various forms of stress destabilize the glutamate system, resulting in significant changes of glutamate release and excitatory transmission in PFC and HPC. In particular, acute inescapable stress is known to increase glutamate release/transmission in PFC, while different forms of chronic stress have been shown to reduce glutamate release/transmission in both PFC and HPC (Musazzi et al., 2010, 2017; Duman and Aghajanian, 2012; Yuen et al., 2012; Kallarackal et al., 2013; Marrocco et al., 2015; Krystal et al., 2017a; Murrough et al., 2017). The functional changes in excitatory transmission are accompanied by changes in neuroarchitecture that are so far well described only for chronic stress and are starting to be unveiled for acute stress (see above). Consequently, in the last several years a major shift has occurred from the traditional monoaminergic hypothesis to a neuroplasticity hypothesis, which takes into account the role of the glutamate system as a mediator of psychiatric pathology (Duman and Aghajanian, 2012; Sanacora et al., 2012; Duman et al., 2016; Murrough et al., 2017; Musazzi et al., 2017).

A major problem here is to understand how the application of stressors, even a short-timed acute stressor, may have long-term consequences. Chronic stress models do not seem to be well-suited to answer this question because the several changes are usually assessed at the endpoint of more or less long periods (days, weeks), which does not tell us about changes occurring in the system along time. In other words, with chronic stress protocols we likely see the resultant of a number of adaptations in the brain and body stress response but learn little about how the whole system reached the endpoint. As an example, if acute stress (at least inescapable stress) induces a sustained activation of glutamate release/transmission in PFC, and instead repeated/chronic stress application results in reduced activation of the same area, we need to be able to understand the dynamics of this biphasic response (Yuen et al., 2012; Tornese et al., 2017). This is further complicated from the evidence that, just like humans, rodents have different ways of responding to stress exposure. Schematically, rodents can often be distinguished in resilient and vulnerable subjects, based on different behavioral, but also cellular and molecular, changes in the long-term stress response (Daskalakis et al., 2013; Nasca et al., 2014; Musazzi et al., 2017).

In many biological phenomena physiological mechanisms may turn into pathological processes. If we assume the difference between physiological and pathological response is also a function of individual vulnerability to stress, our studies should investigate the determinants of resilient versus vulnerable trajectories in the brain stress response. Very little is known of the determinants of different stress response phenotypes, but a better knowledge of these features may help separating different trajectories in the stress response and understand better the pathogenesis of stress-dependent neuropsychiatric disorders.

A possible answer to these problems is a dynamic dissection of the stress response, starting from inescapable acute stress inducing long-term pathology-like phenotypical changes, such as inescapable FS stress that results in PTSD-like behavior in rodents (Musazzi et al., 2017).

## Dynamic Dissection of the Acute Stress Response

We have recently implemented the classical FS stress protocol, which we previously used to assess the changes induced in glutamate synapses and circuitry, in order to answer the following questions:

(1) Is it possible to distinguish resilient and vulnerable animals shortly after the acute stress protocol, as it is done routinely after chronic stress protocols (e.g., chronic mild stress)?
(2) Can such a new protocol be used to identify different trajectories in the individual response to acute stress?

We found recently that rats can be deemed resilient or vulnerable just 24 h after the FS stress protocol, by using a standard sucrose intake (SI) test, as used after chronic mild stress. This test takes advantage of the natural preference of rodents for a sweet, palatable solution, and is routinely used as a test for anhedonic behavior (Christensen et al., 2011). We adapted the SI test to identify FS stress-induced anhedonic phenotype. Baseline SI was established for 5 weeks before the stress protocol was applied. Then, animals were either assigned to FS stress or left undisturbed in their home cages (controls) and SI was measured again before and up to 1 week after acute FS stress. Twenty-four hours after stress, rats showing at least a 25% within-subject decrease in SI were considered anhedonic and classified as *vulnerable* (FS-V), while the others were defined as *resilient* (FS-R). FS-V animals were about half of total stressed rats (Sala et al., 2017; ms. in preparation).

**FIGURE 5:**
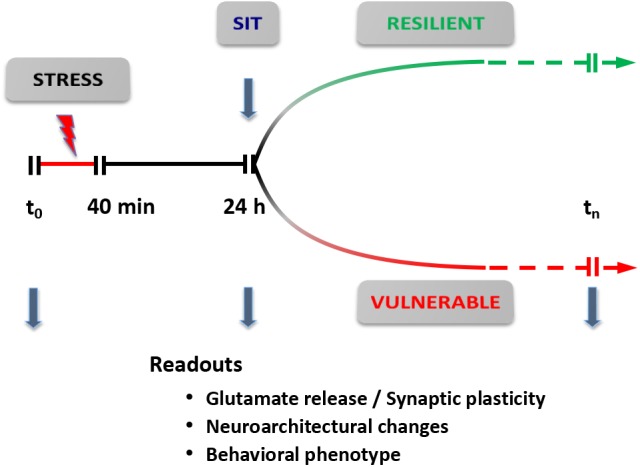
Dynamic dissection of the acute stress response. Conceptual framework for the use of inescapable acute stress (FS stress protocol) to identify different trajectories of resilient versus vulnerable stress response. The readouts are assessed 24 h after start of the stress protocol (40 min). Readouts used are: (i) synaptic plasticity and/or transmission [spontaneous and evoked release of glutamate; spontaneous and evoked excitatory transmission; synaptic plasticity, long-term potentiation (LTP), and/or long-term depression (LTD)]; (ii) neuroarchitectural changes (length and branching of apical dendrites; number of spine synapses); and (iii) behavioral phenotype (SI test for anhedonia; cognitive tests). SIT, sucrose intake test. Modified from Musazzi et al. (2017).

Based on this revised protocol, we are currently carrying out a dynamic dissection of the short- and long-term response to acute stress (**Figure 5**). The rats are subjected to a standard inescapable FS stress protocol (*t*_0_-40 min); 24 h later they are deemed FS-V or FS-R with the SI test, and functional (glutamate release, synaptic transmission), neuroarchitectural (dendritic development, density, and classification of synaptic spines), and behavioral readouts are assessed. These different trajectories can then be followed for longer times to identify behavioral, functional, and neuroarchitectural determinants of the different responses. The identification of molecular and cellular effectors of different trajectories, through accurate dissection of the response and its readouts, may also enable us to identify novel targets for treatment (Musazzi et al., 2017). In addition, this new conceptual framework is also a good way to test rapid-acting antidepressants targeting the glutamate system, in particular ketamine, the prototypic investigational drug in this rapidly expanding field.

## The Ketamine Revolution

Ketamine, a non-competitive antagonist of the *N*-methyl-D-aspartate (NMDA) receptor, has been used as anesthetic since the 1960s, but has also been popular as a recreational drug that induces dissociative behavior and has abuse liability. Since 2000, several clinical studies have shown that a single infusion of low-dose ketamine (tipically 0,5 mg/kg) exerts a rapid (within hours) and sustained (at least a week or longer) antidepressant effect (Berman et al., 2000; Zarate et al., 2006; Murrough et al., 2013). In most studies ketamine was administered to patients with major depression and, in a few cases, with bipolar disorder; a majority of the studies were with treatment-resistant patients. This has been called “arguably the most important discovery in half a century” in psychopharmacology of mood and anxiety disorders (Duman and Aghajanian, 2012). Indeed, the success of ketamine has shown that, by targeting directly the glutamate system, the antidepressant effect can be obtained much faster than with traditional drugs targeting monoaminergic systems. This has not only confirmed that the glutamate system is a primary mediator of psychopathology, but also demonstrated that it is a final common pathway for the therapeutic action of antidepressant agents (Sanacora et al., 2012). However, although a great number of clinical trials with ketamine are currently under way, giving to patients out of controlled studies a drug that induces dissociative behavior still represents a great challenge. As a consequence, several lines of research try to develop drugs that address similar or different target as ketamine in the glutamate system, obtain rapid and sustained antidepressant effect, but do not show the dangerous undesired effects of ketamine (Niciu et al., 2014; Abdallah et al., 2015; Chaki, 2017; Machado-Vieira et al., 2017; Murrough et al., 2017). One of them could be the major ketamine metabolite hydroxynorketamine (HNK), which was reported to exert similar antidepressant action as ketamine, without binding to NMDA receptor and causing the typical dissociative effect of ketamine. It was proposed that HNK works by upregulation of α-amino-3-hydroxy-5-methyl-4-isoxazole propionic acid (AMPA) receptor activity and potentiation of excitatory synapses in mood-relevant brain regions (Zanos et al., 2016). The NMDA receptor-independent action of HNK has been questioned (Suzuki et al., 2017).

A great number of preclinical studies have shown that ketamine exerts rapid and sustained antidepressant effect also in rodents (Maeng et al., 2008; Li et al., 2010; Autry et al., 2011; Koike et al., 2011). This has made ketamine not only a tool to provide an approach for rapid and more efficient antidepressant treatment, but also a paradigm to understand the basic neurobiological underpinnings of stress-related psychopathology (Monteggia et al., 2014; Murrough et al., 2017). Obviously, the mechanism of ketamine has been the subject of intense investigation, and at least two major, not mutually esclusive, cellular/molecular mechanisms have been proposed, that can explain the rapid action of ketamine on neuroarchitecture (Li et al., 2010; Autry et al., 2011). In the first mechanism, ketamine increases expression of synaptic proteins (Arc, PSD-95, GluR1, and synapsin I) in PFC, dependent on rapid and transient activation of mTORC1 signaling, consistent with timing observed for induction of new spine synapses. In the second, ketamine induces rapid BDNF translation in HPC, dependent on reduced phosphorylation and activation of eukaryotic elongation factor 2. Blockade of NMDA receptors is envisaged as the starting point in both mechanisms, although different receptor populations may be involved. The two mechanisms could be present at the same time, because they have been localized to different areas (and different subcellular preparations), and could also be interconnected (because BDNF stimulates mTORC1 signaling). In both cases, activation of AMPA receptors is required for the antidepressant effect of ketamine.

Perhaps the most striking effect of ketamine is the rapid restoration of neuroarchitecture compromised by chronic stress: several studies showed that a single administration of ketamine in just 24 h restores dendritic atrophy/remodeling and loss of synaptic spines induced by chronic stress protocols. This is temporally coincident with the peak of the antidepressant effect, and represents a good validation of the neuroplasticity hypothesis, which posits that structural and functional disruption of glutamate synapes/circuitry, crucial for emotional and cognitive processes, is associated with stress-related psychopathology (Duman and Aghajanian, 2012; Popoli et al., 2012; Sanacora et al., 2012; Sousa and Almeida, 2012; Duman et al., 2016). Traditional antidepressants, such as selective serotonin reuptake inhibitors (SSRIs), need several weeks to elicit restoration of compromised neuroarchitecture.

So far, in most studies published, the effect of ketamine was assessed in the group of stressed animals taken as a whole. Recently, using the sucrose consumption test for anhedonia (see above) we have separated vulnerable from resilient animals during and after a 5-week chronic mild stress protocol, and found that, together with other maladaptive changes, atrophy/remodeling of dendrites was observed only in vulnerable animals. Moreover, we showed that anhedonic behavior, reduction of glutamate release in PFC, dendritic atrophy, and reduced dendritic trafficking of BDNF mRNA were mostly restored in just 24 h by ketamine in vulnerable animals (Tornese et al., 2017; ms. in preparation). These results add to the already known mediators of psychopathology and possible targets of rapid-acting antidepressants.

## Ketamine for PTSD?

The lifetime prevalence of PTSD in the general population is approximately 8%, but it jumps up to much higher figures in war veterans and in any population exposed to traumatic events, like natural disasters, episodes of terrorism, or accidents (Kessler et al., 1995, 2005). Official pharmacotherapy of PTSD is particularly poor if compared to other major psychopathologies: only two drugs are currently approved for therapy by FDA, the two SSRI paroxetine and sertraline. A recent meta-analysis showed that effect sizes for trauma-focused psychotherapies versus active control conditions are greater than medications versus placebo, suggesting that PTSD treatment guidelines need revision (Lee et al., 2016). As a consequence, several other classes of drugs are used in off-label fashion for PTSD treatment in the United States, including anticonvulsants, atypical antipsychotics, hypnotics, and opioids. In order to understand better what pharmacotherapies should be tested for PTSD, a survey was submitted to several PTSD investigators around the world, asking them to rank the top five potential new therapeutic targets for PTSD. The resulting top five mechanisms were: NMDA receptor antagonists, cannabinoid receptor modulators, glucocorticoid receptor agonists, non-SRI antidepressants, and opioid receptor agonists (Krystal et al., 2017b). Therefore, ketamine and rapid-acting glutamatergic drugs are considered the top choice among experimental drugs. A randomized clinical trial tested the efficacy of ketamine in chronic PTSD patients against midazolam, used as active comparator. Ketamine infusion produced significant and rapid reduction in PTSD symptom severity, compared with midazolam, when assessed 24 h after infusion. Ketamine was also associated with reduction in comorbid depressive symptoms and improvement in overall clinical presentation. The study provided the first evidence for rapid reduction in symptom severity following ketamine infusion in patients with chronic PTSD (Feder et al., 2014).

Recently, it was suggested that ketamine may exert a prophylactic action against the effects of stressors, perhaps by enhancing resilience. Mice were administered a single injection of 30 mg/kg ketamine and then 1 week later were subjected to 2 weeks of social defeat (SD) stress, a validated protocol that induces depressive-like behavior. Ketamine-treated mice were protected against the deleterious effects of SD, showing reduced immobility time in the forced swim test. The protective action was not observed when 10 mg/kg ketamine was administered. A similar protective effect of ketamine was found with the LH protocol and after chronic CORT administration. Instead, when ketamine was administered 24 h after conclusion of SD stress, it did not improve depressive-like behavior. The authors concluded that the prophylactic effects of ketamine in stress-related pathology are more robust when the drug is given before stress, with resilience persisting at least 3 weeks postinjection in the SD model and 4 weeks in the CORT model (Brachman et al., 2016). In a subsequent study, they tested the effects of ketamine in a contextual fear conditioning (CFC) protocol (an animal model based on shock-induced conditioned fear), and found that administration of prophylactic ketamine 1 week before, but not after CFC, reduced freezing behavior, facilitating fear extinction. Based on these data the authors suggested there is a limited time window for prophylactic protection and ketamine should be administered before exposure to stressors (McGowan et al., 2017). Further work supports the concept of a prophylactic action of ketamine. Amat et al. (2016) found that 10 mg/kg ketamine, administered 2 h, 1 or 2 weeks before inescapable acute tail shocks, prevented the reduced social investigation in rats and increased extracellular 5-HT levels in basolateral amygdala, typically induced by the stress. Microinjection of ketamine into the prelimbic region of the mPFC replicated the effects of systemic ketamine.

The idea that ketamine may exert a prophylactic action in stress-related psychopathologies, particularly PTSD and major depression, is appealing. A preventative treatment (a sort of vaccine) may apply to different potential risk situations, such as soldiers preparing for war-zone deployment or adolescents with a history of abuse/maltreatment facing the menace of bullism (Price, 2016). However, it would be even more interesting to be able to treat people *after* they have been exposed to traumatic shock, such as in the aftermath of a natural catastrophe. If there was a limited time window for the administration of ketamine, as proposed for the administration of glucocorticoids (Zohar et al., 2011), to reduce the core symptoms of subsequent PTSD, this would represent an attractive alternative for a prophylactic treatment against the onset of psychopathology.

We have recently investigated this possibility in our basic protocol of acute FS stress (so far just using control and stressed animal groups). We know already that previous chronic treatment with antidepressants blocks the typical enhancement of glutamate release/transmission induced in PFC by acute FS. This effect has been linked to antidepressant/anxiolytic action of the drugs (Musazzi et al., 2010, 2013). Our preliminary results showed that a single ketamine injection blocked the stress-induced increase in depolarization-evoked glutamate release if administered either 72 or 24 h, but not 1 or 2 h, before FS stress (10 mg/kg ketamine was injected i.p. at different time points before the FS stress and animals were sacrificed immediately at the end of the 40 min stress protocol). We also found that ketamine completely blocked the increase of depolarization-evoked glutamate release measured 24 h after stress exposure when administered 6 h after FS stress (ms. in preparation). The assessment of related neuroarchitectural changes is currently under way.

Understanding whether and how ketamine can counteract structural and functional alterations induced by acute stress in glutamate synapses and circuitry will be extremely important to find new targets and new strategies to prevent stress-induced maladaptive changes, and possibly triggering a resilient, pro-adaptive stress response.

## Author Contributions

All authors contributed to the design and content of the manuscript, and approved the final version of the manuscript.

## Conflict of Interest Statement

The authors declare that the research was conducted in the absence of any commercial or financial relationships that could be construed as a potential conflict of interest.
